# *ADH1B* and *ALDH2* are associated with metachronous SCC after endoscopic submucosal dissection of esophageal squamous cell carcinoma

**DOI:** 10.1002/cam4.705

**Published:** 2016-03-31

**Authors:** Kenichi Kagemoto, Yuji Urabe, Tomohiro Miwata, Shiro Oka, Hidenori Ochi, Yasuhiko Kitadai, Shinji Tanaka, Kazuaki Chayama

**Affiliations:** ^1^ Department of Gastroenterology and MetabolismHiroshima UniversityHiroshimaJapan; ^2^ Department of Regeneration and Medicine Medical center for Translation and Clinical ResearchHiroshima University HospitalHiroshimaJapan; ^3^ Department of EndoscopyHiroshima University HospitalHiroshimaJapan

**Keywords:** *ADH1B*, *ALDH2*, endoscopy, esophageal squamous cell carcinoma, metachronous

## Abstract

A previous genome‐wide association study identified two novel esophageal squamous cell carcinoma (ESCC) susceptibility genes, *ADH1B* and *ALDH2*. We investigated the characteristics of ESCC, and the relationship between metachronous esophageal and/or pharyngeal squamous cell carcinoma (SCC) and the *ADH1B* & *ALDH2* risk alleles. One hundred and seventeen superficial ESCC patients who underwent treatment with endoscopic submucosal dissection (ESD) were followed up using endoscopy for ≥12 months. First, we performed a replication analysis to confirm the relationship between ESCC and the *ADH1B* & *ALDH2* risk alleles using 117 superficial ESCC cases and 1125 healthy controls. Next, we investigated the incidence and genetic/environmental factors associated with metachronous SCC development after ESD. We also analyzed the potential risk factors for metachronous SCC development using Cox's proportional hazards model. rs1229984 GG located on *ADH1B* and rs671 GA located on *ALDH2* were significantly associated with ESCC progression (*P* = 7.93 × 10^−4^ and *P* = 1.04 × 10^−5^). Patients with rs1229984 GG, those with rs671 GA, smokers, heavy alcohol drinkers (44 g/day ethanol), and presence of multiple Lugol‐voiding lesions (LVLs) developed metachronous SCC more frequently (*P* = 3.20 × 10^−3^, 7.00 × 10^−4^, 4.00 × 10^−4^, 2.15 × 10^−2^, and 4.41 × 10^−3^, respectively), with hazard ratios were 2.84 (95% confidence interval [CI] = 1.43–5.63), 4.57 (95% CI = 1.80–15.42), 4.84 (95% CI = 1.89–16.41), and 2.34 (95% CI = 1.12–5.31), respectively. Multiple logistic regression analysis revealed that rs1229984 GG, rs671 GA, and smoking status were independently associated with the risk of developing metachronous SCCs after ESD. Moreover, we found cumulative effects of these two genetic factors (rs1229984 GG and rs671 GA) and one environmental factor (tobacco smoking) which appear to increase metachrous SCCs after ESD of ESCC risk approximately nearly 12‐fold. Our findings elucidated the crucial role of multiple genetic variations in *ADH1B* and *ALDH2* as biomarkers of metachronous ESCC.

## Introduction

Esophageal cancer is the seventh most common cancer worldwide, and its incidence has increased rapidly over the past three decades 1. There is a marked geographic difference in the incidence and etiology of esophageal cancer. Although esophageal adenocarcinoma is common in Western countries, 80% of esophageal cancers that occur globally are of the squamous cell carcinoma (SCC) type. This SCC type is especially prevalent in Asian countries including China, India, and Japan 2. Most cases of esophageal SCC (ESCC) are diagnosed at advanced stages, with an overall 5‐year survival rate of 10–20%, and often involve the use of modern surgical techniques combined with various treatment modalities 3, 4. In contrast, in a previous study, patients with superficial ESCC (intramucosal or submucosal carcinoma) exhibited an overall 5‐year survival rate of >80% 5.

ESCCs are known to be associated with environmental carcinogens. Heavy alcohol consumption, tobacco smoking, advanced age, and male sex have been reported as risk factors of ESCC 6. Particularly in Japan, tobacco smoking and alcohol consumption were found to be the two major lifestyle factors related with the development of ESCC. The relative risk of developing ESCC is estimated at 3.27 for past smokers and 3.69 for current smokers, respectively, compared with nonsmokers 7. Moreover, alcohol consumption has shown to increase the risk of developing ESCC by 2–3 fold 8.

Advances in endoscopic technology have made it possible to detect ESCCs, which allows for prompt administration of further endoscopic treatment such as endoscopic mucosal resection or endoscopic submucosal dissection (ESD). However, endoscopic treatment spares a larger area of the esophageal mucosa than does surgical resection 9. Accordingly, studies claim that metachronous ESCC develops frequently in patients who have undergone endoscopic treatment 9, 10, 11. Therefore, in order to prevent death due to metachronous SCC, endoscopy‐based biomarkers and surveillance programs are necessary.

Recently, genome‐wide association studies (GWAS) have been widely used for the analyses of disease susceptibility genes 12, 13, 14, 15, 16. In 2009, Cui et al. conducted a GWAS of the Japanese population and identified a strong association between ESCC and variants of the rs1229984 and rs671 alleles on the *ADH1B* and *ALDH2* genes 17.

In this study, we investigated the utility of two single‐nucleotide polymorphisms (SNPs) located on *ADH1B* and *ALDH2* as biomarkers of metachronous ESCC and/or pharyngeal SCC after ESD for ESCC.

## Patients and Methods

### Patients

We enrolled 217 patients with esophageal dysplasia/ESCC who consented to providing samples between December 2012 and December 2014. We excluded patients with advanced ESCC (*n* = 24), history of esophageal disease (*n* = 4), low‐grade intraepithelial neoplasia (*n* = 8), granular cell tumors (*n* = 2), and those without follow‐up for >12 months (*n* = 51). Furthermore, nine patients with ESCC who did not undergo ESD and two patients who underwent esophageal surgery after ESD were excluded. Finally, a total of 117 patients (101 male, 16 female; mean age: 64.7 years) were enrolled in this study, including 103 patients with superficial ESCC and 14 with high‐grade intraepithelial neoplasia (HGIN). Ninety‐five patients (81%) underwent curative resection by ESD according to the Japanese esophageal cancer treatment guidelines 18, and 10 patients underwent chemoradiotherapy (CRT) after ESD, and the remaining patients were observed without additional surgical resection. Follow‐up surveillance endoscopy was performed 12 months after the procedure and once every 12 months thereafter. The median observation period was 38.8 months (range: 13–128 months).

Metachronous tumors were defined as ≥1 primary tumors detected distant from the ESD scar. Tumors detected in close proximity to the scar were regarded as local recurrent tumors. We obtained 1125 samples, which served as healthy controls, from volunteers at the Hiroshima University, Hiroshima, Japan. We used the recommended Lugol spraying method to detect ESCCs. Because squamous dysplasia and SCCs lack glycogen, these lesions are known as Lugol‐voiding lesions (LVLs) 9, 19, 20. During endoscopic examination, Lugol's solution was sprayed via a catheter. A speckled LVL pattern was defined as much more than 10 small LVLs or numerous irregularly shaped multiform LVLs in the esophageal mucosa 21.

### Evaluation of clinicopathologic features

We investigated the incidence of metachronous tumors in 117 patients using the Kaplan–Meier method and retrospectively investigated the clinicopathologic features associated with metachronous tumors, including alcohol consumption, smoking, multiple LVLs, history of CRT, rs1229984, and rs671. We also evaluated the outcomes of metachronous tumors after ESD.

In patients with synchronous multiple tumors, we chose, as the main lesion, a tumor with the highest malignant potential as determined by the presence of a malignancy, diffuse type disease, or increased tumor size or depth. Tumor location and macroscopic type were classified according to the Japanese Classification of Esophageal Carcinoma 22. The pathological diagnosis of each tumor was also determined according to the Japanese Classification of Esophageal Carcinoma criteria 22.

### Two SNPs on *ALDH2* and *ADH1B* genotyping

Genomic DNA was extracted from peripheral blood leukocytes using a standard method. We genotyped 117 cases and 1125 healthy controls by using a multiplex PCR‐based Invader assay (Third Wave Technologies).

### Statistical analyses

The association between the SNPs and ESCC risk using replication analysis was tested with logistic regression analysis adjusted for age and sex by assuming allelic, dominant, recessive, and overdominant models using the JMP statistical software package (SAS Institute Inc., Cary, NC). The odds ratios (ORs) were calculated using the nonsusceptible allele as a reference, unless stated otherwise. The cumulative incidence of metachronous ESCCs/HGIN was evaluated using the Kaplan–Meier method. To analyze the potential risk factors, such as age (0 = 60 years/<60 years; 1 = >60 years), sex (0 = female, 1 = male), rs671 at *ALDH2* (0 = AA+GG, 1 = GA), rs1229984 at *ADH1B* (0 = AA+AG, 1 = GG), alcohol consumption (0 = never or light drinker, 1 = heavy drinker), smoking (0 = never smoker, 1 = smoker), multiple LVLs (0 = none or <10 small LVLs, 1 = many more than 10 small LVLs or numerous irregularly shaped multiform LVLs 21, and CRT after ESD (0 = none, 1 = treatment); for metachronous tumors, we performed univariate analysis using the Kaplan–Meier method, log‐rank test, and Cox's proportional hazards modeling. On the basis of the median level of alcohol consumption (44 g/day) for a regular alcohol drinker, the population was categorized into two classes: nondrinkers or light drinkers (0–44 g/day) and heavy drinkers (>44 g/day). Multiple logistic regression analysis was used to assess the contributions of confounding factors with the JMP statistical software package (SAS Institute Inc), and the following explanatory variables were included in the analysis: rs671 at *ALDH2*, rs1229984 at *ADH1B*, alcohol consumption, and smoking. A *P*‐value of <0.05 was considered significant. Cox's proportional hazards model was used to estimate the hazard ratio and 95% confidence interval (CI).

## Results

In the replication analysis to confirm the relationship between superficial ESCC and the *ADH1B* & *ALDH2* risk alleles, we genotyped 117 superficial ESCC cases and 1125 healthy controls using invader assay (Table S1). The relationship between superficial ESCC and the *ADH1B* rs1229984 and *ALDH2* rs671 alleles are shown in Table 1. Among the superficial ESCC patients, 36 (30.8%) had the ALDH2 rs671 allele characterized by the GG genotype, 80 (68.4%) had the GA genotype, and 1 (0.9%) had the AA genotype. Furthermore, of the patients with the *ADH1B* rs1229984 allele, 31 (26.5%) had the GG genotype, 36 (30.8%) had the GG genotype, and 50 (42.7%) had the AA genotype. The following was observed in the healthy controls: *ALDH2* rs671: GG, 642 cases (57.1%); GA, 432 cases (38.4%); AA, 51 cases (4.5%) and *ADH1B* rs1229984: GG, 60 cases (5.3%); GA, 389 cases (34.6%); AA, 676 cases (60.1%). Replication analysis with an Armitage‐trend model revealed that rs671 and rs1229984 were significantly associated with superficial ESCC (*P* = 5.62 × 10^−5^ and 1.65 × 10^−9^, OR = 1.75 and 2.43, respectively; data not shown). We found a remarkable difference between the cases and controls in terms of sex and age, and logistic regression analyses confirmed age and sex as significant covariates.

**Table 1:** The effect of genetic factors on the risk of developing ESCC

SNP	Location	Allele	ESCC patients				Healthy controls				Allelic model 1 versus 2		Dominant model 11 + 12 versus 22		Recessive model 11 versus 12 + 22		Overdominant model 12 versus 11 + 22	
	Gene	1/2	11	12	22	MAF	11	12	22	MAF	*P*	OR (95%CI)	*P*	OR (95%CI)	*P*	OR (95%CI)	*P*	OR (95%CI)
rs671	12q24	G/A	36	80	1	0.35	642	432	51	0.24	8.61 × 10^−4^	2.02	5.18 × 10^−5^	3.99	0.72	1.14	1.04 × 10^−5^	4.28
	*ALDH2*		0.31	0.68	0.01		0.57	0.38	0.05			(1.3–3.14)		(2.00–7.97)		(0.14–9.04)		(2.18–8.39)
rs1229984	4q23	G/A	31	36	50	0.42	60	389	676	0.23	1.35 × 10^−3^	2.05	2.35 × 10^−2^	1.55	7.93 × 10^−4^	3.94	0.43	0.83
	*ADH1B*		0.26	0.31	0.43		0.05	0.35	0.60			(1.32–3.2)		(0.8–3.03)		(1.76–8.86)		(0.57–1.23)

A total of 117 ESCC patients and 1125 controls were analyzed.

*P* ‐value was obtained using logistic regression analysis adjusted for age and sex.

ORs and CIs were calculated using the nonsusceptible alleles as a reference.

MAF, minor allele frequency; SNP, single‐nucleotide polymorphisms; ESCC, esophageal squamous cell carcinoma; OR, odds ratio; CI, confidence interval.

Futhermore, we analyzed two SNPs, rs671 on *ALDH2* and rs1229984 on *ADH1B*, using allelic, dominant, recessive, and overdominant models adjusted for age and sex.

We found that rs1229984 on *ADH1B* showed the strongest association with superficial ESCC in the recessive model (allelic model: *P* = 1.35 × 10^−3^, OR = 2.05, 95% CI = 1.32–3.2; dominant model: *P* *=* 2.35 × 10^−2^, OR = 1.55; 95% CI = 0.8–3.03; recessive model: *P* *=* 7.93 × 10^−4^, OR = 3.94, 95% CI = 1.76–8.86). Consistent with previous reports 17, we found that rs671 exhibited the strongest association with development of superficial ESCC in the overdominant model (allelic model: *P* *=* 8.61 × 10^−4^, OR = 2.02, 95% CI = 1.32–3.14; dominant model: *P* = 5.18 × 10^−5^, OR = 3.99, 95% CI = 2.0–7.97; recessive model: *P* *=* 7.17 × 10^−1^, OR = 1.14, 95% CI = 0.14–9.04; overdominant model: *P* *=* 1.04 × 10^−5^, OR = 4.28, 95% CI = 2.18–8.39).

Next, we investigated the incidence of metachronous SCC after ESD in 117 patients with esophageal tumors using the Kaplan–Meier method (Fig. S1–8). Sample characteristics are showed in Table 2. Thirty‐four patients developed metachronous SCC (15 SCCs and 19 HGINs), and the median period until discovery after initial ESD was 39.9 months (range: 12–101 months). According to the investigation of initial/metachronous tumors, three patients had HGINs/HGINs, 19 had SCCs/HGINs, and 12 had SCCs/SCCs, respectively. The cumulative incidence curve of metachronous esophageal tumors revealed a gradual increase and an incidence of 10.4% per year.

**Table 2:** Characteristics of samples obtained from 117 patients after ESD

ESCC (metachronous/without metachronous)	34/83
Sex (Male/Female)	101/16
Multiple LVLs (+/−)	99/18
Heavy alcohol consumption (+/−)	63/54
Smoking (+/−)	83/34
CRT (+/−)	10/107
rs671 at *ALDH2* (GA/GG+AA)	80/37
rs1229984 at *ADH1B* (GG/GA+AA)	31/86

LVLs, Lugol‐voiding lesions; ESCC, esophageal squamous cell carcinoma; CRT, chemoradiotherapy.

To further validate the incidence of metachronous SCC development after ESD in association with genetic/environmental factors (age, sex, presence of multiple LVLs, alcohol consumption, smoking status, history of CRT for ESCCs treated with ESD, *ALDH2* rs671*, ADH1B* rs1229984; Table S2). Presence of multiple LVLs, heavy alcohol consumption, smoking, rs671 GA, and rs1229984 GG significantly affected the incidence of metachronous tumors on the basis of the univariate analysis performed using the Kaplan–Meier method and log‐rank test (*P* = 3.58 × 10^−1^, 2.46 × 10^−2^, 1.20 × 10^−3^, 1.70 × 10^−3^, and 1.72 × 10^−3^, respectively; Fig. S1–8). These associations persisted after adjustment using the Cox proportional hazards model. The hazard ratios were as follows: heavy alcohol consumption, 2.34 (95% CI = 1.12–5.31); smoking, 4.84 (95% CI = 1.89–16.41); *ALDH2* rs671 GA, 4.57 (95% CI = 1.80–15.42); and *ADH1B* rs1229984 GG, 2.84 (95% CI = 1.43–5.63; Table S2). Multivariate analysis revealed that *ADH1B* rs1229984 GG, *ALDH2* rs671 GA, and smoking status were independently associated with the risk of developing metachronous SCCs after ESD (Table 3).

**Table 3:** Multiple Cox's proportional hazards analysis for the risk factors of metachronous SCC after ESD

Risk factor	Metachronous cases	Without metachronous cases	Hazard ratios	95% CI	*P*‐value
Multiple LVLs	34	65			0.09
Heavy alcohol consumption	25	38	1.35	0.64–3.13	0.45
Smoking	30	53	3.38	1.28–11.68	1.19 × 10^−2^
*ALDH2*; rs671 GA	30	50	3.28	1.28–11.15	1.08 × 10^−2^
*ADH1B*; rs1229984 GG	17	14	2.21	1.10–4.45	2.59 × 10^−2^

Herein, 34 patients with metachronous SCC and 83 patients without metachronous SCC after endoscopic resection were analyzed.

All patients with metachronous SCC had multiple LVLs.

Hazard ratios and CIs were calculated using the nonrisk environmental factors and nonsusceptible allele as a reference.

LVLs, Lugol‐voiding lesions; CI, confidence interval; CRT, chemoradiotherapy; SCC, squamous cell carcinoma.

**Figure 1:**
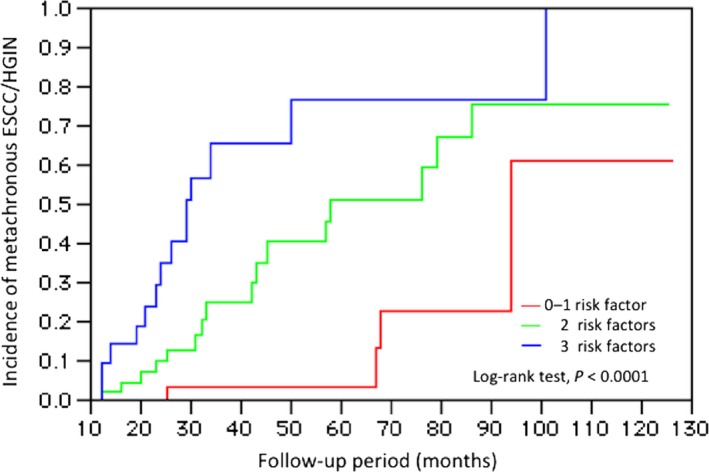
The cumulative incidence of metachronous ESCCs/HGIN in 117 patients with ESCC who underwent treatment with ESD, according to the number of independent risk factors (*ADH1B* rs1229984 GG,*ALDH2* rs671 GA, and smoking) ESCC, esophageal squamous cell carcinoma; HGIN, high‐grade intraepithelial neoplasia.

We also examined the additive effect of two SNPs and smoking (Table 4 and Fig. 1) on the development of metachronous SCCs after ESD. We found that the risk of metachronous SCC after ESD increased 4.56‐fold (95% CI = 1.66–15.9) in patients with two risk factors and 11.95‐fold (95% CI = 4.21–42.69) in patients with three risk factors, which was higher than that in patients with none or one risk factors; these findings therefore indicate the cumulative effects of these variants on metachronous SCC susceptibility.

**Table 4:** Cox's proportional hazards analysis for the risk of metachronous SCCs after ESD according to the number of risk factors

Number of risk factors	Metachronous cases	Without metachronous cases	Hazard ratios	95% CI	*P* ‐value
0–1	4	42	1		
2	16	34	4.56	1.66–15.9	2.3 × 10^−3^
3	14	7	11.95	4.21–42.69	<1.0 × 10^−3^

Herein, 34 patients with metachronous SCC and 83 patients without metachronous SCC after endoscopic resection were analyzed.

The three risk factors are *ADH1B* rs129984GG allele, *ALDH2* rs671 GA allele, and smoking.

CI, confidence interval; SCC, squamous cell carcinoma, ESD, endoscopic submucosal dissection

## Discussion

Alcohol consumption has shown to increase the risk of developing various types of cancer 8, but pure ethanol was not found to act as a carcinogen in animal studies 23. Acetaldehyde, a primary metabolite of ethanol, is considered to be a plausible candidate with carcinogenic effects; in fact, acetaldehyde inhalation was shown to induce various types of tumors, particularly adenocarcinoma and SCC of the nasal mucosa, in animal models 24, 25. The ethanol in alcohol is metabolized to acetaldehyde by alcohol dehydrogenase‐1B (*ADH1B*), and the acetaldehyde is metabolized to acetate by aldehyde dehydrogenase‐2 (*ALDH2*). *ADH1B* is located at 4q23, which encodes the beta subunit of class I alcohol dehydrogenase (ADH), an enzyme that catalyzes the rate‐limiting step for ethanol metabolism: the oxidation of alcohol to acetaldehyde. *ALDH2* is located at 12q24.2, which encodes a member of the alcohol dehydrogenase family. Members of this enzyme family metabolize a wide variety of substrates, including ethanol, retinol, other aliphatic alcohols, hydroxysteroids, and lipid peroxidation products. This encoded protein, consisting of several homodimers and heterodimers of alpha, beta, and gamma subunits, exhibits high activity for ethanol oxidation and plays a major role in ethanol catabolism. The *ADH1B* and *ALDH2* genes contain SNPs that modulate enzymatic activity. A previous GWAS identified two novel ESCC susceptibility genes: *ADH1B* (rs1229984) and *ALDH2* (rs671) 17, 26. A nonsynonymous SNP (rs1229984) generates two allelic variants: ADH1B*1 (Arg48, G213) and ADH1B*2 (His48, A213). ADH1B*2 (A) was reported to exhibit 30–40‐fold higher enzymatic activity for ethanol oxidation than ADH1B*1 (G) 27. A nonsynonymous SNP (rs671) also generates two allelic variants: ALDH2*1 (Glu504, G1951) and ALDH2*2 (Lys504 A1951). ALDH2*2 (A) allele encodes a catalytically inactive subunit. ADH1B*1 (G) and ALDH2*2 (A) are prevalent genotypes found in approximately 90% and 50% of populations in East Asian countries such as Japan, China, and Korea 28. In this study, we selected superficial esophageal SCC cases treated with ESD, and performed a replication study to confirm the relationship between ESCC and the *ADH1B* & *ALDH2* risk alleles by using an Invader assay. *ADH1B* rs1229984 GG allele and *ALDH2* rs671 GA allele were found to be risk factors of ESCC.

The incidence of metachronous SCC after ESD was estimated at 29% in this study, which is higher than that previously reported (12–15%) 10, 29. However, a study did claim that metachronous ESCC occurred in 35% of alcoholic patients after endoscopic resection. In that report, *ALDH*2*1/*2 was found to be the risk factor of metachronous ESCC 11. In this study, the proportion of ESCC patients with the *ALDH2* GA allele was 68.4%, which may explain the high incidence of metachronous ESCC. The median interval to the detection of a second tumor after the initial ESD was 39.9 months (range: 12–101 months), and the Kaplan–Meier curve seemed to reach a plateau after 100 months.

Despite some previous studies wherein metachronous ESCC occurred more frequently in patients with the speckled LVL pattern in the background mucosa compared with patients without LVLs^9^, ^30^, LVLs was not found to be a risk factor for metachronous SCC in this study. This may be attributable to the advances in endoscopic technology such as magnifying endoscopy. We can detect early‐stage lesions more easily by using white light endoscopy and narrow band imaging before using the Lugol spraying method.

*ADH1B* rs1229984 GG, *ALDH2* rs671 GA, and smoking status, but not heavy alcohol consumption, were independently associated with the risk of developing metachronous SCCs after ESD in this study. We believe that this is due to the genetic risk factors: rs1229984 GG allele and rs671 GA allele had a stronger influence and closer association, which would have diminished the effects of alcohol consumption. However, this study included only a few patients with metachronous SCC, which made a detailed analysis difficult. Further studies involving more patients are needed. Moreover, the study was retrospective in nature and conducted at only a single center. Future studies should be aimed at a prospective analysis in multiple centers.

Several previous GWAS have reported SNPs associated with disease incidence; however, most SNPs are not used in clinical pathology. This study elucidated the crucial role of two SNPs, identified using a GWAS in the *ADH1B* & *ALDH2* genes, as biomarkers of metachronous SCC after ESD in superficial ESCC. ESD for superficial ESCC successfully improved the survival rate. However, metachronous SCCs occurred highly frequently after ESD. Therefore, the estimation of metachronous SCC risk after ESD for superficial ESCC would be essential to guide personalized treatment and achieve optimal results. In this study, we developed a risk model for metachronous SCCs using genetic and environmental factors. We found that individuals in the highest risk category have a nearly 12‐fold higher risk of developing metachronous SCCs than those in the lowest risk category. We are confident that our findings will greatly contribute to the establishment of personalized surveillance for superficial ESCCs after ESD. Our findings elucidated the crucial role of multiple genetic variations in *ADH1B* and *ALDH2* as biomarkers of metachronous ESCC.

## Conflict of Interest

The authors declare no conflict of interest associated with this manuscript.

## Supporting information


**Figure S1.** The cumulative incidence of metachronous ESCC/HGIN in 117 patients with ESCC who underwent treatment with endoscopic submucosal dissection, according to age. ESCC, esophageal squamous cell carcinoma; HGIN, high‐grade intraepithelial neoplasia.

**Figure S2.** The cumulative incidence of metachronous ESCCs/HGIN in 117 patients with ESCC who underwent treatment with endoscopic submucosal dissection, according to sex. ESCC, esophageal squamous cell carcinoma; HGIN, high‐grade intraepithelial neoplasia.

**Figure S3.**The cumulative incidence of metachronous ESCCs/HGIN in 117 patients with ESCC who underwent treatment with endoscopic submucosal dissection, according to the presence of multiple LVLs. ESCC, esophageal squamous cell carcinoma; HGIN, high‐grade intraepithelial neoplasia; LVLs, Lugol‐voiding lesions.

**Figure S4.**The cumulative incidence of metachronous ESCCs/HGIN in 117 patients with ESCC who underwent treatment with endoscopic submucosal dissection, according to alcohol consumption. ESCC, esophageal squamous cell carcinoma; HGIN, high‐grade intraepithelial neoplasia.

**Figure S5.**The cumulative incidence of metachronous ESCCs/HGIN in 117 patients with ESCC who underwent treatment with endoscopic submucosal dissection, according to smoking status. ESCC, esophageal squamous cell carcinoma; HGIN, high‐grade intraepithelial neoplasia.

**Figure S6.**The cumulative incidence of metachronous ESCCs/HGIN in 117 patients with ESCC who underwent treatment with endoscopic submucosal dissection, according to history of CRT. ESCC, esophageal squamous cell carcinoma; HGIN, high‐grade intraepithelial neoplasia; CRT, chemoradiotherapy.

**Figure S7.**The cumulative incidence of metachronous ESCCs/HGIN in 117 patients with ESCC who underwent treatment with endoscopic submucosal dissection, according to the presence of the ALDH2 rs671 genotype. ESCC, esophageal squamous cell carcinoma; HGIN, high‐grade intraepithelial neoplasia.

**Figure S8.**The cumulative incidence of metachronous ESCCs/HGIN in 117 patients with ESCC who underwent treatment with endoscopic submucosal dissection, according to the presence of the ADH1B rs1229984 genotype. ESCC, esophageal squamous cell carcinoma; HGIN, high‐grade intraepithelial neoplasia.

**Table S1.** Characteristics of samples and methods used in this study.

**Table S2.** Cox's proportional hazards analysis for the risk factors of metachronous SCCs after ESD.
