# Supplementary figures and images for: ADH1B and ALDH2 are associated with metachronous SCC after endoscopic submucosal dissection of esophageal squamous cell carcinoma
Source: Cancer Med. 2016 Mar 31;5(7):1397–404. doi: 10.1002/cam4.705 (PMC4944865; doi:10.1002/cam4.705)

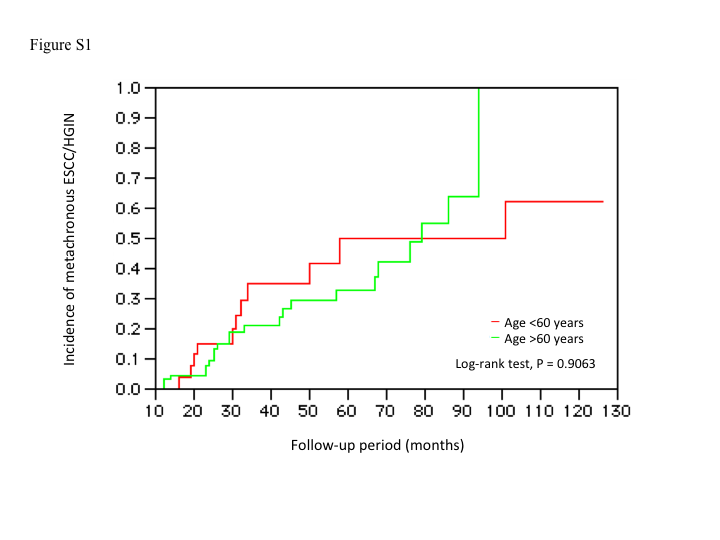

Supplement: Supplementary file 1 — Figure S1. The cumulative incidence of metachronous ESCC/HGIN in 117 patients with ESCC who underwent treatment with endoscopic submucosal dissection, according to age. ESCC, esophageal squamous cell carcinoma; HGIN, high‐grade intraepithelial neoplasia. [file CAM4-5-1397-s001.tiff]

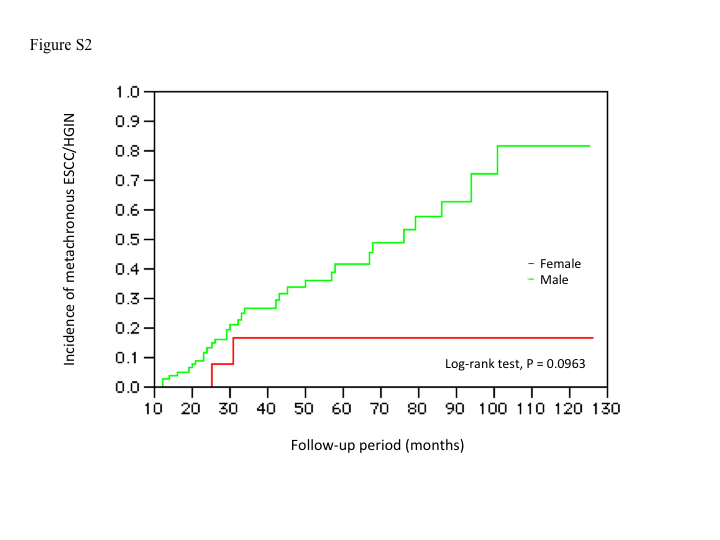

Supplement: Supplementary file 2 — Figure S2. The cumulative incidence of metachronous ESCCs/HGIN in 117 patients with ESCC who underwent treatment with endoscopic submucosal dissection, according to sex. ESCC, esophageal squamous cell carcinoma; HGIN, high‐grade intraepithelial neoplasia. [file CAM4-5-1397-s002.tiff]

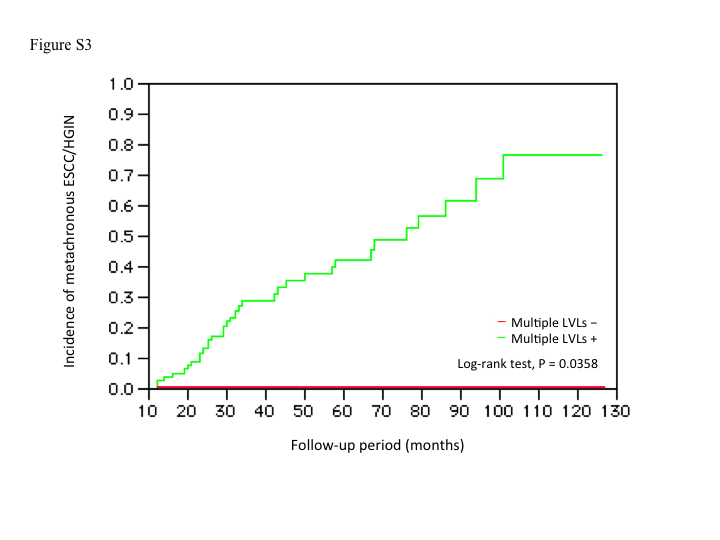

Supplement: Supplementary file 3 — Figure S3.The cumulative incidence of metachronous ESCCs/HGIN in 117 patients with ESCC who underwent treatment with endoscopic submucosal dissection, according to the presence of multiple LVLs. ESCC, esophageal squamous cell carcinoma; HGIN, high‐grade intraepithelial neoplasia; LVLs, Lugol‐voiding lesions. [file CAM4-5-1397-s003.tiff]

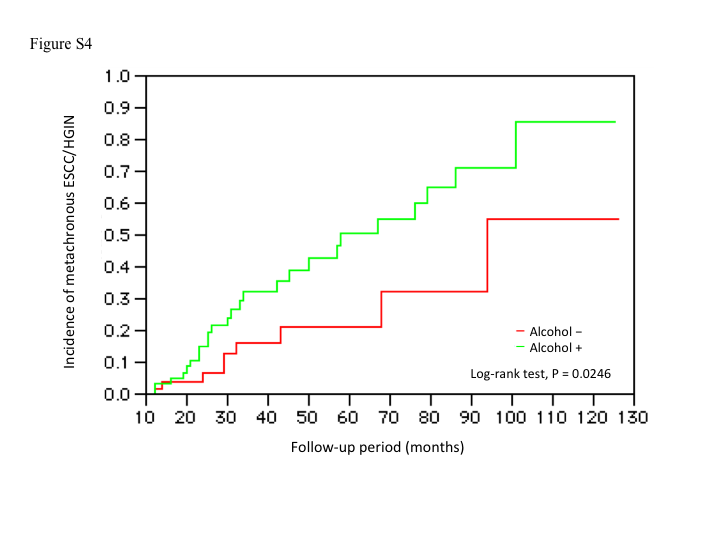

Supplement: Supplementary file 4 — Figure S4.The cumulative incidence of metachronous ESCCs/HGIN in 117 patients with ESCC who underwent treatment with endoscopic submucosal dissection, according to alcohol consumption. ESCC, esophageal squamous cell carcinoma; HGIN, high‐grade intraepithelial neoplasia. [file CAM4-5-1397-s004.tiff]

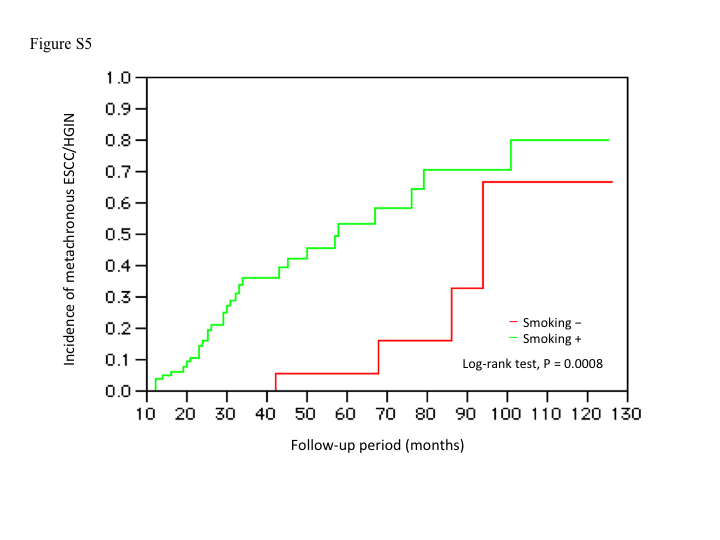

Supplement: Supplementary file 5 — Figure S5.The cumulative incidence of metachronous ESCCs/HGIN in 117 patients with ESCC who underwent treatment with endoscopic submucosal dissection, according to smoking status. ESCC, esophageal squamous cell carcinoma; HGIN, high‐grade intraepithelial neoplasia. [file CAM4-5-1397-s005.tiff]

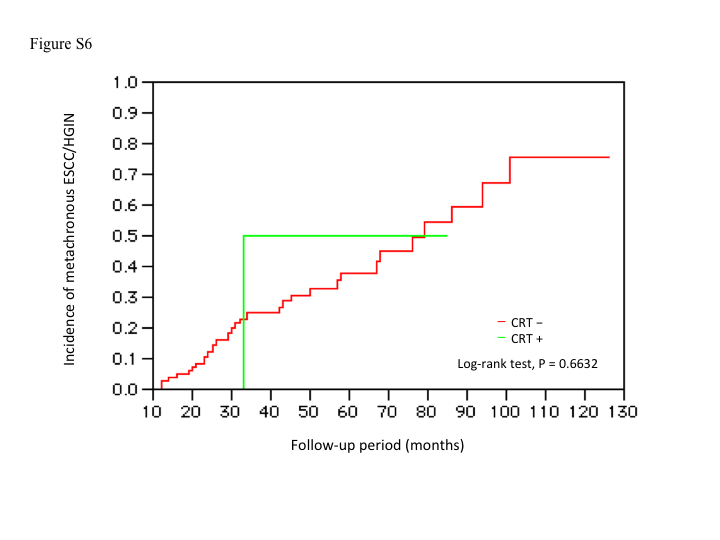

Supplement: Supplementary file 6 — Figure S6.The cumulative incidence of metachronous ESCCs/HGIN in 117 patients with ESCC who underwent treatment with endoscopic submucosal dissection, according to history of CRT. ESCC, esophageal squamous cell carcinoma; HGIN, high‐grade intraepithelial neoplasia; CRT, chemoradiotherapy. [file CAM4-5-1397-s006.tiff]

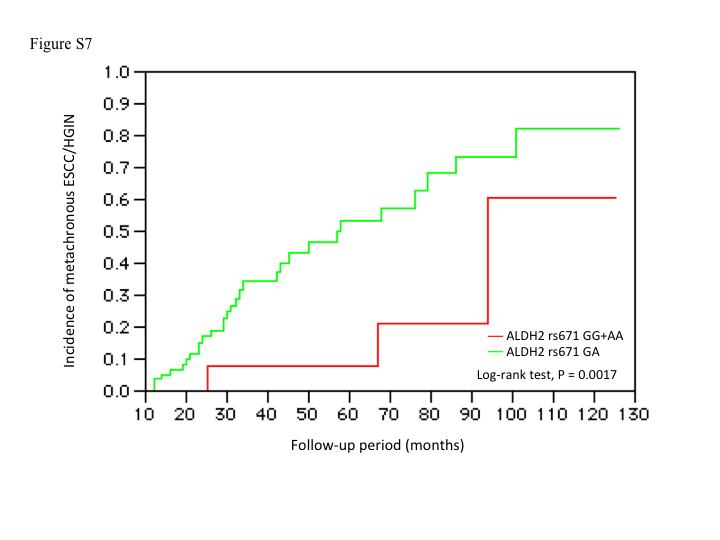

Supplement: Supplementary file 7 — Figure S7.The cumulative incidence of metachronous ESCCs/HGIN in 117 patients with ESCC who underwent treatment with endoscopic submucosal dissection, according to the presence of the ALDH2 rs671 genotype. ESCC, esophageal squamous cell carcinoma; HGIN, high‐grade intraepithelial neoplasia. [file CAM4-5-1397-s007.tiff]

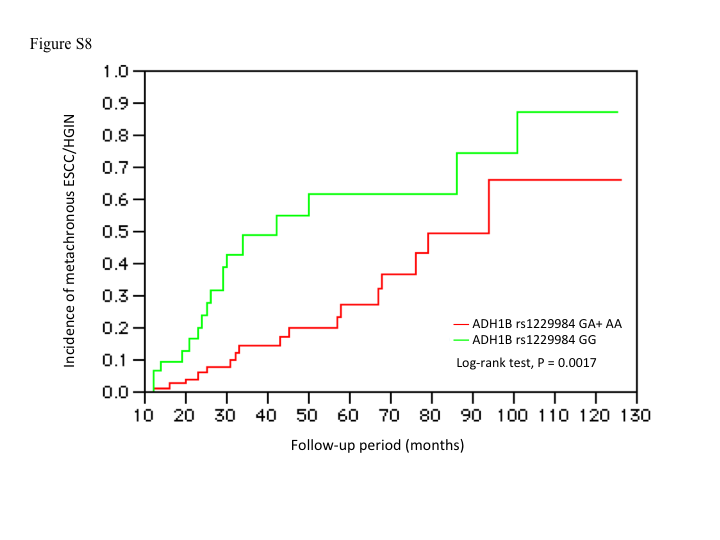

Supplement: Supplementary file 8 — Figure S8.The cumulative incidence of metachronous ESCCs/HGIN in 117 patients with ESCC who underwent treatment with endoscopic submucosal dissection, according to the presence of the ADH1B rs1229984 genotype. ESCC, esophageal squamous cell carcinoma; HGIN, high‐grade intraepithelial neoplasia. [file CAM4-5-1397-s008.tiff]
