# Supplementary material for: ADH1B and ALDH2 are associated with metachronous SCC after endoscopic submucosal dissection of esophageal squamous cell carcinoma
Source: Cancer Med. 2016 Mar 31;5(7):1397–404. doi: 10.1002/cam4.705 (PMC4944865; doi:10.1002/cam4.705)
Supplement: Supplementary file 9 — Table S1. Characteristics of samples and methods used in this study. [file CAM4-5-1397-s009.docx]

Table S1. Characteristics of samples and methods used in this study

| Sample | Source | Platform | Number of samples | Female (%) | Age (mean ± SD) |
| --- | --- | --- | --- | --- | --- |
| ESCC | Hiroshima University | Invader assay | 117 | 16 (14.3%) | 64.7 ± 8.92 |
| Control | Hiroshima University | Invader assay | 1125 | 694 (61.7%) | 41.5 ± 15.2 |

Control samples were obtained from healthy volunteers.

All subjects with a history of malignancy were excluded from the control group.

ESCC, esophageal squamous cell carcinoma; SD, standard deviation
