# Supplementary material for: ADH1B and ALDH2 are associated with metachronous SCC after endoscopic submucosal dissection of esophageal squamous cell carcinoma
Source: Cancer Med. 2016 Mar 31;5(7):1397–404. doi: 10.1002/cam4.705 (PMC4944865; doi:10.1002/cam4.705)
Supplement: Supplementary file 10 — Table S2. Cox's proportional hazards analysis for the risk factors of metachronous SCCs after ESD. [file CAM4-5-1397-s010.docx]

Table S2. Cox's proportional hazards analysis for the risk factors of metachronous SCCs after ESD

| Risk factor | Metachronous cases | Without metachronous cases | Hazard ratios | 95%CI | p-value |
| --- | --- | --- | --- | --- | --- |
| Age (> 60 years) | 23 | 67 | 0.95 | 0.46-2.14 | 0.9 |
| Male sex | 32 | 69 | 3.15 | 0.95-19.47 | 0.06 |
| Multiple LVLs | 34 | 65 |  |  | 4.41×10^-3^ |
| Heavy alcohol consumption | 25 | 38 | 2.34 | 1.12-5.31 | 2.15×10^-2^ |
| Smoking | 30 | 53 | 4.84 | 1.89-16.41 | 4.00×10^-4^ |
| CRT | 6 | 13 | 1.25 | 0.46-2.88 | 0.62 |
| *ALDH2*; rs671 GA | 30 | 50 | 4.57 | 1.80-15.42 | 7.00×10^-4^ |
| *ADH1B*; rs1229984 GG | 17 | 14 | 2.84 | 1.43-5.63 | 3.20×10^-3^ |

LVLs, Lugol-voiding lesions; CI, confidence interval; CRT, chemoradiotherapy; SCC, squamous cell carcinoma

Herein, 34 patients with metachronous SCC and 83 patients without metachronous SCC after endoscopic resection were analyzed.

All patients with metachronous SCC had multiple LVLs.

Hazard ratios and CIs were calculated using the non-risk environmental factors and non-susceptible allele as a reference.
